# Male support for family planning and contraceptive use by their partners during the COVID-19 pandemic in selected states in Nigeria: a qualitative explorative study

**DOI:** 10.1186/s12978-025-02230-8

**Published:** 2026-01-02

**Authors:** Tanimola M. Akande, Adesola Oluwafunmilola Olumide, Abiodun S. Adeniran, Oloruntomiwa Oyetunde, Taiwo Gboluwaga Amole, Maryam Jimoh, Olufemi A. Fawole, Babatunde Oluwagbayela, Komal Preet Allagh, Rita Kabra

**Affiliations:** 1 University of Ilorin, Ilorin, Nigeria; 2 Institute of Child Health, University of Ibadan, Ibadan, Nigeria; 3 Bayero University, Kano, Nigeria; 4 University of Ilorin Teaching Hospital, Ilorin, Nigeria; 5 UNDP/UNFPA/UNICEF/WHO/World Bank Special Programme of Research, Development and Research Training in Human Reproduction (HRP), Department of Sexual, Reproductive, Maternal, Child, Adolescent Health and Ageing, World Health Organization, 20 Avenue Appia, Geneva, 1211 Switzerland

**Keywords:** Male involvement, Male partner support, Contraception, Family planning, COVID-19, Nigeria, WHO

## Abstract

**Background:**

The COVID-19 pandemic affected all facets of life, including access to health and other social services. The World Health Organization conducted a multi-country mixed methods study in India, Nigeria and Tanzania to assess the impact of the pandemic on family planning (FP) access, and the health system's capacity to provide FP and contraceptive services. In this paper, we share results of the qualitative aspect of the study that explored men’s support for FP and contraceptive use by their partners during the COVID-19 pandemic in Nigeria.

**Methods:**

A qualitative exploratory study was conducted in rural and urban communities in Kano, Kwara, and Oyo states of Nigeria among women of reproductive age and their male partners. One hundred and forty-seven women and 95 male partners were purposively selected, and they participated in 68 in-depth interviews and 21 focus group discussions. The interviews and discussions were digitally recorded and subsequently transcribed. Data were organized using ATLAS.ti and analysed using content analysis.

**Results:**

The mean age of the respondents was 34.4 ± 10.9 years, 90% were married, and 74% had at least secondary education. In Kano, decisions regarding FP and contraceptive use were majorly made by men alone, whereas in Kwara and Oyo states, decisions were often made jointly by the couple. The other forms of male support reported were largely comparable in the three study states. For example, communicating with their female partners about FP, providing practical support such as financial assistance and transportation, accompanying their partners to health facilities or drug stores to procure contraception, offering physical support during the procedure and emotional support. Use of contraceptive methods by men, which is a direct form of support was not frequently reported.

**Conclusions:**

Males played notable roles in supporting their partners to obtain FP and contraception during the pandemic. We recommend implementing interventions that encourage greater male involvement in FP, as well as interventions that promote joint decision-making between couples in settings where decisions are mostly taken by men alone.

## Plain language summary

Family planning (FP) offers numerous benefits for women, families and communities, yet its utilization remains low in Nigeria. Male partners play a significant role in influencing FP and contraceptive uptake among women in Nigeria and other developing countries. During the COVID-19 pandemic, efforts to control the spread of the virus affected health service provision and access, including FP and contraceptive access. In this paper, we present findings from our study that examined men’s support for FP access and use by their female partners during the pandemic. We carried out interviews and discussions with 147 women aged 18 – 49 years and 95 male spouses and partners in Kano, Kwara and Oyo states. In Kano, decisions regarding FP use were primarily made by men alone, whereas in Kwara and Oyo, decisions were often made jointly by couple or by the women alone. Many men in the study states supported their wives/partners who needed FP during the pandemic by discussing the need for FP with them, seeking information about facilities where FP methods were available, providing funds for FP methods and driving their partners to facilities to obtain FP services. These findings highlight the importance of programmes that promote men’s support for FP use in enabling women to get FP and contraceptives during emergencies.

## Background

Although contraceptive prevalence rates among women of reproductive age in sub-Saharan Africa rose from 13 percent in 1990 to 29 percent in 2019, the utilization of modern contraceptive methods remains low compared to other regions [1]. In Nigeria, the use of modern contraceptives was reported as 14 percent in 2018 [2]. This indicates that women still face significant barriers in seeking and accessing family planning (FP) information and services [3]. Various factors at the individual, family and societal levels influence the utilization of modern FP among women of childbearing age [4]. One significant factor in many low- and middle-income countries is the patriarchal system, where men are the decision makers including in issues relating to health, FP, and contraceptive use [3–6]. Thus, male involvement in FP plays a crucial role in FP and contraceptive service uptake in these countries.

Male involvement in contraceptive use refers to, “all organizational activities aimed at men as a discrete group which have the objective of increasing the acceptability and prevalence of family-planning practice of either sex” [7]. It covers male utilization of a family planning method and actions that promote partner utilization of a method [8, 9]. Male involvement is further categorized as direct involvement – use of a contraceptive method by males, and indirect involvement, such as discussions and support for partner utilization of FP and contraceptive methods. Studies report that indirect male involvement is more commonly practiced than direct involvement [10, 11]. Interventions that engage men and boys in FP programmes therefore aim to enhance their participation in FP and contraceptive use, foster better communication and decision-making between partners, and encourage men to advocate for gender equality and promote FP within their families and communities [12].

Galle et al. (2021) developed a global multidimensional male involvement framework to facilitate the assessment and comparison of male involvement in maternal health, which encompasses five elements namely, communication, decision-making, practical, physical, and emotional involvement [13]. Although this framework was originally designed for maternal health, we adapted it for the context of family planning, which is an integral aspect of maternal health.

Prior to the COVID-19 pandemic, interventions aimed at enhancing FP and contraceptive uptake in Nigeria, including those targeting male support for FP, have been implemented. For example, a key objective of Nigeria's National Family Planning Communication Plan (2017–2020) was to attain a 50% coverage rate of couples engaging in discussions about FP issues by the end of 2018. Planned Parenthood Federation of Nigeria (PPFN) implemented educational campaigns targeting community, traditional and religious leaders, government officials and other male groups following which an increase in male support for FP was observed [7]. The Nigerian Urban Reproductive Health Initiative (NURHI) implemented from 2009 to 2015 focused on service provision, demand generation, advocacy, monitoring and evaluation, and resulted in an increased modern contraceptive method use by men [14]. Findings from the NURHI-2 evaluation survey also revealed that 68% of the people who listened to the “Go Men Go” radio programme dedicated to promoting male involvement in FP, engaged in FP discussions with their spouses within six months of the evaluation [15].

Following the declaration of COVID-19 as a pandemic and the subsequent implementation of control measures including lockdowns, there were notable disruptions in health service provision, especially for non-emergency services such as FP and contraceptive services. Factors influencing male involvement in FP and contraceptive use, such as access to media and spousal employment status [16], were also affected by the pandemic. In addition, movement restrictions resulted in spouses and partners being together at home for longer periods, which may have led to higher sexual activity, thus creating a higher need for FP and contraception [17, 18]. While various studies have reported on FP demand, utilization and service provision during the COVID-19 pandemic, there is a paucity of studies reporting the role of men in supporting FP and contraceptive use by their partners during this period.

**Fig. 1:**
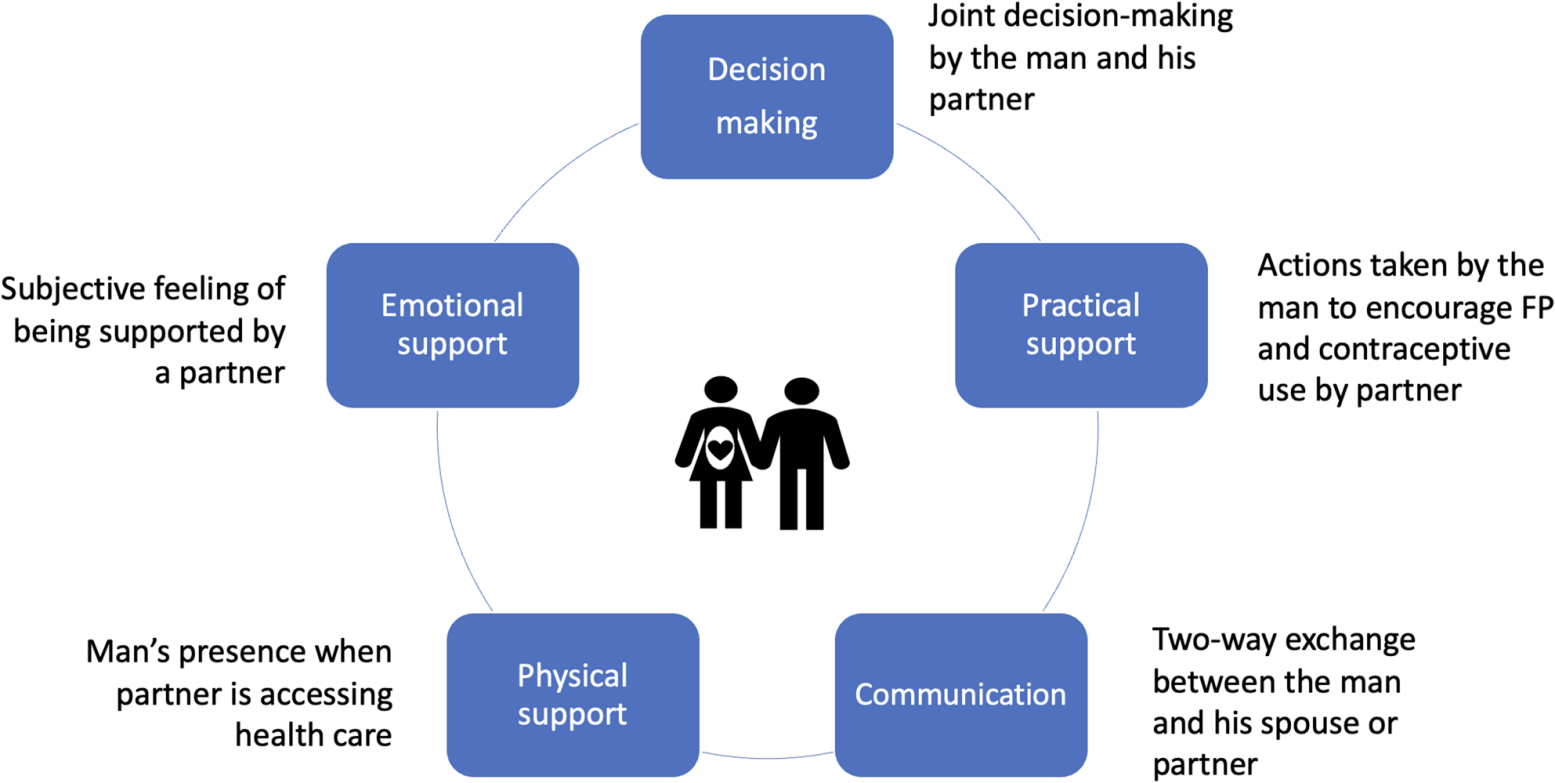
Global framework for assessing male involvement in maternal health. Adapted from Galle et al. [13]

In the current paper, we explored men’s support for family planning and contraceptive use by their female partners during the COVID-19 pandemic in three selected states in Nigeria. This was part of findings from a larger mixed-method study commissioned by the World Health Organization (WHO), Human Reproduction Program (HRP) in 2021 to determine health facility readiness to provide FP services and evaluate the barriers to availability, utilization and readiness of FP and contraceptive services in India, Tanzania, and Nigeria. The study protocol and detailed methodology have been reported in a previous publication [19]. We adapted the framework on male involvement in maternal health described by Galle, Griffin and Osman et al. (2021) [13] for the analysis (as depicted in Fig. 1). Our study findings may inform interventions aimed at encouraging male support for FP and contraceptive use, especially during situations that call for movement restrictions as occurred during the COVID-19 pandemic.

## Methods

### Study design

A qualitative exploratory study was conducted in Kano, Kwara and Oyo states, Nigeria and information was obtained via in-depth interviews (IDIs) and focus group discussions (FGDs).

### Setting

The study was conducted in rural and urban communities in Kano, (Northwest geo-political zone) Kwara (Northcentral) and Oyo state (Southwest geopolitical zone); (Table 1). Data collection took place in May and June 2022 when healthcare and other services had reopened. The study locations were purposively selected for the following reasons: (i) to provide some geographical spread (ii) comparable multi-level Sexual and Reproductive Health (SRH) and FP interventions sponsored by the Bill and Melinda Gates Foundation namely, NURHI (Oyo and Kwara) and The Challenge Initiative (TCI) (Kano) had been implemented in the states and (iii) the lead in-country investigator had pre-existing professional networks within these states.

**Table 1:** Contextual information during the period of the COVID-19 pandemic* by state

**State**	**Geopolitical zone**	**FP intervention**	**Contraceptive prevalence rate**	**Total number of COVID-19 cases by August 2021 **[20]	**COVID-related movement restrictions**
Kano - Dala Urban LGA - Kura Rural LGA	Northwest	TCI	6.3%	4,029	A two-week lock down was imposed in addition to closure of non-essential services
Kwara -Ilorin West (Urban LGA) - Afon Asa (Rural) LGA	Northcentral	NURHI	22.4%	3,235	Movement restrictions (but no lockdown) and closure of non-essential services
Oyo - Ibadan North (Urban LGA) - Akinyele (Peri-urban LGA)	Southwest	NURHI	22.6%	7,143	Movement restrictions (but no lockdown), and closure of non-essential services

^*^This covered the period February 2020 when the first COVID-19 case was reported up until June 2022 when the data was collected

Two Primary Health Care (PHC) facilities were selected per state – one in a rural or peri-urban location and another in an urban location. Facilities providing maternal and child health and FP services, with high volume of clients were purposively selected.

### Characteristics of participants

Study participants comprised female community members of reproductive age (18 to 49 years), female clients of reproductive age who visited the selected PHCs for FP and contraceptive services and their male partners. All participants were required to express a willingness to participate and provide written informed consent. The following were excluded from participating:

- Females who could not wait to be interviewed, for example those referred to other facilities if their preferred contraceptive method was not available in the PHC, or women referred for additional health care
- Male partners who were unable to be interviewed e.g. those unable to come to the facility because they were out of town at the time the interviews were conducted
- Participants who did not provide consent

### Sample size

For the pilot, one FGD each among female clients, male partners and female community members, and two IDI each among female clients and their partners were planned. For the main study, we estimated that we would conduct a maximum of 18 FGDs (six FGDs with female community members: two FGDs per state, six FGDs with female clients accessing FP and contraceptive services, and six FGDs with their partners), and 48–72 in-depth-interviews (24—36 with female clients and 24–36 with their male partners). We anticipated that with these numbers we would attain saturation. We however planned to conduct additional FGDs and IDIs if new information was being obtained until saturation was attained. A total of 21 FGDs and 68 IDIs (34 females and 34 male partners) were eventually conducted (See breakdown in Table 2).

**Table 2:** Distribution of participants by facility and data collection approach

Facilities	Client in facility		Community
	Focus group discussions		
2 PHC in each state (3 states)	Female Number of FGD conducted (estimated number)	Male Number of FGD conducted (estimated number)	Female Number of FGD conducted (estimated number)
Kano	2 (2)	2 (2)	2 (2)
Kwara	2 (2)	2 (2)	2 (2)
Oyo	2 (2)	2 (2)	2 (2)
Total FGDs (Main study)	6 (6)	6 (6)	6 (6)
Oyo (pilot)	1 (1)	1 (1)	1 (1)
Total FGDs (pilot and main study)	7 (7)	7 (7)	7 (7)
	In-depth interviews		
	Female Number interviewed (estimated sample)	Male Number interviewed (estimated sample)	
Kano	10 (8–12)	10 (8–12)	N/A
Kwara	10 (8–12)	10 (8–12)	
Oyo	12 (8–12)	12 (8–12)	
Total – IDI (Main study)	32 (24—36)	32 (24—36)	
Oyo (pilot)	2 (2)	2 (2)	
Total – IDI (pilot and main study)*	34 (26–38)	34 (26–38)	
Total (IDIs and FGDs) conducted	34 IDIs 7 FGDs	34 IDIs 7 FGDs	7 FGDs

^*^including pilot

### Instruments

In-depth interview guides for female clients, and their male partners were used to obtain information on interviewee socio-demographic characteristics, decision making and agency to act with respect to FP. The male IDI guide further sought information on the role men played in ensuring their partner sought and/or accessed the needed FP and contraception services. The FGD guides for female clients, their male partners and female community members included questions that enquired about access to FP services during the pandemic. The guides were translated into Yoruba, the predominant language in Oyo and Kwara states, and Hausa in Kano state. The guides were back-translated to English to ensure accuracy, and following a review by research staff, the final versions used on the field were arrived at.

### Data collection procedure

Health facility staff, acting as gatekeepers, assisted in recruiting the female clients, after they obtained the intended services. The clients were informed about the study and those willing to participate were referred to the project manager for the informed consent process. Participation was voluntary, and only those providing informed consent were enrolled and interviewed. For participants interested in the FGDs, contact information was collected to enable the project manager reach out to them after a convenient date was agreed on by potential participants. The female clients were also asked if their male partners could be contacted as possible participants. Those who agreed provided their partners’ phone number and this was shared with the project manager who subsequently called the partner, introduced the study and enquired if he would like to participate. Male partners who agreed to participate were taken through the screening questionnaire on phone and asked to come to the health facility on a convenient day for the IDI or FGD. All interviews and FGDs were conducted in a quiet room in the facility where participant privacy and confidentiality were assured.

Health facility and community gatekeepers assisted with recruitment of females of reproductive age from within the community. Interested females were invited to the health facility for the FGD, after undergoing the informed consent process. A brief questionnaire on socio-demographic characteristics was administered to all FGD participants before commencement of the discussion.

Qualified research assistants who conducted the IDIs and FGDs were recruited and trained to ensure the validity of the data collection and to protect privacy and confidentiality of the participants. To ensure comfort and privacy of participants, female researchers conducted the interviews with women, while male researchers conducted interviews with men. The IDIs lasted about 40 min, while FGDs lasted from 50 to 90 min.

After each interview and FGD, research assistants sent the recording to the team supervisor who stored them securely for transcription. To protect the data from unintended use, the audio files were encrypted and sent to the data coordination center. Only members of the research team were authorized to access the recordings, which were retained until transcription and accuracy checks were completed.

A pilot study was conducted in Ibadan, Oyo State prior to the main study to ensure the validity of the questions and identify any issues with the data collection process which would require revision prior to the main study. We conducted three FGDs: one among eligible women accessing FP in the health facility, one among their male partners and a third among community women not accessing FP in the health facility. Two IDIs were conducted among women accessing FP and two among male partners. Data from the pilot was incorporated into the main study as there were no major revisions to the study instrument after the pilot. Our ethical approval included the pilot and informed consent was obtained from pilot participants.

For the main study, three FGDs were conducted per facility: one FGD with women (18–49 years) accessing FP services in the health facility, one FGD among their male partners and one FGD among women in the community. In addition, in-depth interviews were conducted with women accessing FP from the health facility and their partners. We endeavoured to purposively enroll female clients who are more likely to be missed, e.g., younger female clients ages 18 to 24 years and female clients with disabilities, to participate in the IDIs.

Despite a significant reduction in COVID-19 transmission and the lifting of mask mandates during the data collection period, safety precautions were still implemented to protect the study team. These measures included encouraging the team members to wear masks in enclosed spaces with limited ventilation, selecting training venues with adequate ventilation, and providing hand sanitizer for both research staff and participants.

### Ethics

The generic research protocol was approved by WHO RP2 and WHO Ethics and Research Committee (Project no. A66007). Adapted country-specific protocols received ethical approval from National Health Research Ethics Committee of Nigeria – NHREC/01/01/2007–07/09/2021, University of Ilorin Ethical Research Committee and the State Ministries of Health research ethics committees in Kano, Kwara and Oyo States. Informed consent was obtained from each participant before conducting the interviews and discussions. Participants who agreed to participate signed the consent form. Participants who were not literate provided verbal consent. The participants were informed that participation was voluntary, they could choose to withdraw from the study at any stage or skip any questions, they do not feel comfortable responding to, without any negative consequences.

### Statistical analysis

The interviews and FGDs were analysed following the general approach of content analysis. This involved reading through the verbatim transcriptions and notes to gain an understanding of what was being expressed by the participants, noting down emerging themes and patterns in the data and condensation of the data [21]. Codebook creation was informed by MaCQueen et al. [22]. The initial version of the codebook was shared with the study team for review and feedback. Two coders independently applied the codebook to a few transcripts and then discussed for harmonization. Additional sub-codes were developed as required. The transcripts were then distributed among a team of coders who coded independently. The coding leads subsequently reviewed each transcript with the coders and where there were discrepancies, these were discussed and revised. During transcription of the interviews and discussions, all identifying data were removed, guaranteeing participant anonymity. The recordings were destroyed after the data had been transcribed and reviewed by the site coordinators.

For the current paper, three members of the team reviewed the transcripts, discussed the findings specific to the current paper and developed additional sub-codes where required. Analysis followed both an inductive and deductive approach. The analysts then conducted a cross-case analysis to explore similarities or differences in responses to the relevant questions by gender, rural–urban location and state.

## Results

Sixty-eight in-depth interviews and 14 focus group discussions with women and male partners were conducted in health facilities providing FP services, and seven FGDs were conducted with women in communities in rural and urban LGAs in Kano, Kwara and Oyo states. Overall, two hundred and forty-two respondents (147 women and 95 men) participated in the IDIs and FGDs. The participants mean age was 34.4 years and half had education up to secondary level (Table 3). Fourteen participants, comprising twelve females and two males refused to participate.

**Table 3:** Socio-demographic characteristics of study participants

Socio-demographic characteristics	Rural (%) *n* = 113	Urban (%) *n* = 129	Total (%) *n* = 242
Age (years)			
≤ 19	0 (0%)	9 (7%)	9 (4%)
20—35	77 (68%)	72 (56%)	149 (62%)
> 35	36 (32%)	48 (37%)	84 (35%)
Mean age (± SD)	34.4 (11.7)	34.3 (10.3)	34.4 (10.9)
Sex			
Female	65 (57%)	82 (64%)	147 (61%)
Male	48 (43%)	47 (36%)	95 (39%)
Education Level			
None	2 (2%)	15 (12%)	17 (7%)
Primary (1–5 years)	28 (25%)	19 (15%)	47 (19%)
Secondary (6–9 years)	55 (49%)	66 (51%)	121 (50%)
High school-Vocational school	19 (17%)	8 (6%)	27 (11%)
University	8 (7%)	19 (15%)	27 (11%)
Graduate school	1 (1%)	2 (2%)	3 (1%)
*Mean number of school years achieved (*± *SD)*	*10.1 (3.6)*	*9.1 (4.6)*	*9.6 (4.2)*
Marital Status			
Married/Cohabiting	113 (100%)	128 (99%)	241 (99%)
Separated	0 (0%)	1 (1%)	1 (0.5%)
Monthly Income (USD)^1^			
< 100	92 (81%)	106 (82%)	188 (78.7%)
100–199	17 (15%)	17 (13%)	37 (15.5%)
200–399	3 (3%)	4 (5%)	8 (3.3%)
> 399	1 (1%)	0 (0%)	6 (2.5%)
Median (IQR)	48.2 (57.8)	48.2 (55.5)	(56)

^1^CBN exchange rate as at 7th July 2022 1USD = 414.83NGN (https://www.cbn.gov.ng/rates/exchratebycurrency.asp)

The qualitative data findings are presented under the following themes: (i) Female perspectives about decision-making for FP and contraceptive use, (ii) Male perspectives about decision-making for FP and contraceptive use by female partner and (iii) Male involvement in supporting female partners to access needed FP during the pandemic.

### Female perspectives about decision-making for FP and contraceptive use

The women interviewed indicated that decisions regarding the uptake of a contraceptive method were made either solely by their husbands or partners, jointly as a couple, individually by themselves, or occasionally by other family members. Female perspectives about decision-making for FP and contraceptive use varied depending on the study location. In Kano, nearly all women, regardless of whether they resided in rural or urban areas, reported that their male partners were primarily responsible for deciding whether or not they use a contraceptive. They explained that although they sometimes expressed their desire to adopt a particular method to their husbands, the ultimate decision rested with their husbands.

*“No, I did not because my husband did not ask me to do it. “Even now, that I am using contraceptives, it is his instructions I am following”* (KN_IDI_FPU_03_URBAN)

“It is my husband that makes the decision. All I need is just to ask him that, I will like to continue, and he makes the decision”.(KN_IDI_FP_02-RURAL)

“My husband, because I am under his control, so he dictates all that I do” (KN_IDI_FP_02_URBAN)

While majority of female interviewees in Kano mentioned that their husbands were the decision-makers, one woman residing in the urban community in Kano mentioned that, for her, this was always a joint decision, “*It is a joint decision always. I always present the topic and we decide together*”.

In contrast, in Kwara and Oyo states, decisions regarding contraceptive use were mainly joint decisions, with few women stating that their husbands made the decision independently. Additionally, women in Kwara and Oyo reported instances where they made the decision independently, a situation less frequently observed in Kano.

“We do it together, husband and wife” (FGD with female FP users, rural community, Kwara)

“… all I can say is, me and my husband decide the space [number of years we want] between our children”. (Female FP user, IDI, urban community, Kwara)^.^

“We can also decide to go by ourselves” (Female FP users, rural community in Kwara, FGD)

A female FP user residing in a rural area in Kwara explained that the husband typically makes the decision regarding FP. However, she took the decision herself due to the complications she experienced during childbirth.

*“The person that makes FP decision is the husband but because I as the wife have experienced obstructed labour and much childbirth, I also informed my husband that I wish to do family planning. (Female FP user (IDI), rural community, Kwara)*

*“After I had my second child, I told myself I was going to wait a while before having another. That’s why I did it. I took the decision on my own. I told my husband and he gave his consent”* **(***Female FP user (IDI), rural community, Oyo)*

*“I am responsible for the decision, I am the one who will be pregnant and carry the pregnancy, I am the one who knows if I can take care of a child or not. No one can tell me when to take up a family planning or not, it is the number of children you have that will enter your room, you must plan your life” (Female FP user (IDI), rural community, Oyo)*

According to a female FP user in Oyo, she and her husband made the decision jointly, although she was the one who went to get the method. “*I was the only one that went*” *( Female FP user, rural community, Oyo).*

### Male perspectives about decision-making for FP and contraceptive use by female partner

Similar to the female respondents, perspectives of the males about decision-making for FP and contraceptive use also varied by location, with men in Oyo and Kwara being more likely to mention joint decision-making, unlike most men in Kano, who were often the sole decision-makers. However, some men in Kano also reported making decisions jointly with their wives. The men confirmed that decisions regarding FP and contraceptive needs were typically made either by them alone or jointly with their female partners. It was uncommon for men to mention that the decision-making rested solely with the woman.

*“I am the one responsible for carrying out the final decision to take up the family planning because I have other responsibilities of my relatives, parents and my siblings that is why I don’t want to have more children”. (Male partner IDI, urban community, Kano)*

*“I am the only person, as the husband, to decide what my wife will do because my parents are late [dead] and whatever she is trying to do she must consult me before she carries it out”. (Male partner IDI, urban community, Kano)*

*“When she went to the family unit [in the hospital] she called me to tell me that there are different methods and there is implant for 2–3 years. Then I told her to insert the one for three years duration. So, she took the Implanon for 3 years duration” (Male partner IDI, rural community, Kano)*

*"In the first place, it is the husband that makes the decision related to FP and contraceptives needs in the family” (Male partner IDI, rural community, Kano)*

In addition to being the primary decision maker, a participant in Kano took the initiative to inquire about FP methods, following which both he and his wife visited the facility, where she obtained a method.

*“… you see without that spacing, the child, the mother and the unborn child will all have difficulties. This is what actually convinced me for my wife to have some rest of about two to three years. I came to the facility and asked the health workers for information on family planning. They told me that I have to come with my wife and there is a form that we need to fill. But they said the good thing about it is that as the husband, I have agreed. We went and were told about it and chose a method. Up to today, we are practicing it (Male partner, FGD participant, rural community, Kano).*

A male participant from an urban community in Kano said, “… *both of us decided to take up the FP and contraceptive*”. Another male participant, also from Kano, made a joint decision with his wife to take up an FP method due to complications during her last pregnancy. He explained, *“Yes, both of us decided to take up family planning because she had suffered during her last delivery”.* In Kwara, a male partner from the rural community expressed, “*When I say it [bring up the issue of family planning], my wife agrees, and we go and do it together”.*

A male partner in Oyo state explained how he and his wife made a joint decision*, “… listening to each other—both myself and my wife. When we reach a conclusion about it, we go to the hospital for counselling on what to do. The healthcare workers are best to tell which of the options is good for us”* (*Male partner, IDI, rural community, Oyo)*. 

In the same vein, another male respondent residing in the urban community in Oyo shared, “*The two of us are supposed to have an agreement about the family planning and collectively make a decision, not that only the wife will make the decision without informing the husband, this can scatter the home”.*

In Oyo, some men also decided to purchase and use a condom as their preferred FP method *“I am the one that makes the decision because I am the bread winner of the home, so I have to think of how we don’t bear too many children we cannot cater for and train. So, it [is] my responsibility” (IDI male participant urban Oyo).*

Some men in the rural community of Oyo expressed support for FP as they wanted to have a manageable family size because of the economic implications of having too many children. However, they were concerned about the potential side effects, such as delayed conception after discontinuing a method, weight gain and perceptions of increased promiscuity among women. They urged the government to ensure that the methods were truly reversible, allowing women to conceive when desired once they discontinue the method.

Some expressed concerns about covert use and recommended that the government addresses this issue. They suggested that health workers should only provide FP services if the couple visited the facility together and only after ascertaining that they were indeed a couple.

*“There are some wives that will take another man to [the facility] for family planning so that they can be cheating. Let our government work on it so that when you want to do family planning, they can check if both of you are truly a couple or not” (Male FGD participant, rural community, Oyo state)*

*“They [the couple who desire to take up family planning] can show evidence that they are husband and wife, so that they can arrange the family planning to be solid”. (Male FGD participant, rural community Oyo state)*

### Male involvement in supporting female partners to access needed FP during the pandemic

Not all male participants mentioned that their wives needed FP during the pandemic. However, for those whose partners required it, their involvement in supporting their female partners to obtain the needed FP and contraception services during the pandemic are reported under sub-themes that align with the key elements of male involvement. These elements including communication, decision-making, physical, practical and emotional support, are described in the Global framework for assessing male involvement in maternal health by Galle A, et al [13].

#### Communication about FP

Communication as a component of male involvement should involve discussions with one’s partner. The responses from the male participants included phrases like, “talking to” their partner and engaging in discussions with them.

*“So, I talked to my wife about family planning, and she agreed to do it; then we came here and we chose the injectable method and she is now on family planning”. (Male partner, IDI, urban community, Kano)*

A male participant in Oyo mentioned that despite the lack of discussion between him and his spouse, she did not choose a contraceptive method due to the belief that contraceptives could cause weight gain, which his wife wanted to avoid.

*“No, the reason she didn’t do it is because we heard it makes women add weight and she’s big, so [she] doesn’t want to add extra weight, though we didn’t talk about doing FP or not. Then some people use that [family planning] as an opportunity to be promiscuous” (*Male partner, IDI, urban community, Oyo)

#### Decision-making about FP during the pandemic

Most of the participants were already using an FP method and had made the decision to do so before the pandemic. However, during the pandemic, decisions revolved around where to obtain a method for new users or the need to switch methods due to unavailability or side effects experienced with previous methods.

Men’s involvement in FP decision-making during the pandemic largely mirrored pre-pandemic practices. In Kano, men predominantly made the decisions, while in Kwara and Oyo, decisions were mainly joint among respondents residing in both rural and urban communities as outlined below.

***“*** *The role I played to ensure my partner sought and accessed the needed FP contraceptives was that she asked for consent and I permitted her to take up the services”. (Male partner, IDI, urban community, Kano)*

A male partner in Kwara explained, *“my wife and I [make the decision together], because we know how well we want to train our children*”

A participant in Oyo stated that he had to persuade his wife as she was initially not interested in getting a method, *“… she does not even want to answer [discuss about FP] but I did not keep quiet [did not stop bringing up the issue]”.* (*Male partner, IDI, rural community, Oyo)*

#### Practical involvement of male-partners

Practical actions by men to enable their partners to obtain FP included initiatives such as gathering information about the availability of FP commodities and sharing it with their partners, reminding them to get the chosen method, making transportation arrangements, and providing financial support for obtaining FP methods.

*“And if you have a motorcycle, you can carry your wife to the clinic and at times we have a permit card which I used to show to the authority on the road when they stop us”* (*Male partner, FGD, urban community, Kano)*

“*There was a time the injectable became unavailable, during the COVID-19 pandemic, but because I am close to the health workers, I asked them if they have and that was how we got it”. (Male partner, IDI, rural community, Kano)*

*“The role I played was that during that time I listened to the news and heard about family planning. I went to the health center to confirm after which I told my spouse to go to the health facility we have confirmed for family planning uptake depending on which she prefers because there are different types for different people. So they will explain to her, test her and do it for her”. (Male partner, FGD, urban community, Kwara)*

*“I took her to hospital for treatment and contraceptive service myself” – (Male partner, IDI, Kwara)*

*“We didn’t change it [the family planning method]. I took her [to the facility] on a motorbike then. There was even one day I was taking her, and policemen stopped us that, “weren’t we told not to go out?”, When I explained to them, they told us to go”. (Male partner, IDI, rural community, Kwara)*

And in Oyo, “*I make sure I remind her when it’s time”.* (*Male partner, IDI, rural community, Oyo)*

In Oyo, when the agreed method was a condom, the men ensured this was available for use.

*“I ensure condoms are always available for use when we need it, that’s my responsibility” (Male partner, IDI, urban community, Oyo)*

Men in Oyo and Kwara also discussed additional ways in which they supported their wives, for example, setting up timely reminders and providing financial support.

*“I make sure I remind her when its time. If there’s financial support, maybe to transport her to where she’s going to … I inspect the expiry date of the contraceptive. Even when I am not around, I will snap [take a picture of the information on] the card so that I can follow up”. (Male Partner, IDI, Oyo)*

“If she needs money, I give it to her so she can go.” (Male partner, IDI, Kwara)

#### Physical involvement of male partners

Some participants who drove their partners to health facilities to get a contraceptive method, sometimes waited until they were attended to, demonstrating their supportive role. A male participant in Oyo mentioned, “*I drove her there. I waited outside for her while they attended to her* …” (Male partner (IDI), rural community, Oyo).

Similarly, a male partner in the urban community in Kwara reported that he accompanied his wife to get their preferred method (injectable), *“… we went to the hospital to get family planning service.”*

#### Emotional involvement

Emotional involvement refers to a subjective feeling of being supported by a partner. Although participants did not explicitly mention giving or receiving emotional support, some of their responses hinted at emotional involvement. In Kano (urban community), a female FP user shared that she always initiated discussions about FP use, and her husband supported her, *“It is a joint decision always. I always present the topic, and we decide together … my husband is the one that decides about family planning”.*

Meanwhile, in Oyo, a male partner demonstrated sensitivity to his wife’s feelings by purchasing condoms, as she felt shy buying them in their community. He narrated “… *the only challenge was that only few shops were opened then*, so my wife tells me *that I should go and get the condom that she is shy about buying it in the community. That’s the only challenge”.*

“*…I tell her that if she is coming from work and she does not buy the condom, there is no way we can both have sex but when I saw that there was lockdown, when I close from work, I go to a place far from my community to get the condom in large amount so we can be using it.”*

When asked why he felt shy since he was married, he explained, *“You know then; when you go and buy it in the pharmacy in your community, they will say you are wayward, that you have a wife at home and you are coming to buy condom, so because of our Yoruba culture, it’s better to go and buy it from a far place to avoid this”.*

In Oyo, a female partner shared that her husband discussed with her the need for them to support each other by planning their family. She explained *“My husband wants us to support each other, our child has started school. My husband is a salary earner. Now that they've [my husband’s employers] not paid, they [the school authorities] will chase the kid from school. Then, feeding in the house and everything. That's why he said that I should go for family planning to delay the second pregnancy.”* She later explained that her husband took the lead on this decision and subsequently paid for her to receive an FP method.

## Discussion

We explored the roles that men played in supporting family planning and contraceptive use by their female partners during the COVID-19 pandemic in selected states in southern and northern Nigeria. The role of male partners in supporting FP and contraception use is evolving, particularly in patriarchal societies like Nigeria [23]. With sub-optimal contraceptive rates in Nigeria, and the significant disruptions caused by the COVID-19 pandemic, understanding the roles played by male partners to support FP and contraceptive use by their partners is crucial.

Our findings revealed that husbands were largely identified as the primary decision makers in Kano and in a few instances, in Kwara and Oyo. This reflects the patriarchal structure prevalent in Nigeria, where decision-making in the household is often assigned to husbands [23]. This structure appeared more pronounced in Kano compared to both rural and urban areas of Kwara and Oyo states, where joint decision-making about FP use was prevalent. Unlike Kano, instances were also observed in Kwara and Oyo, where the wife or female partner played the dominant role in decision-making regarding FP and contraception. Various reasons may explain this disparity, including the higher levels of education, increased autonomy of women in North central and Southern parts of Nigeria compared to the core north (Kano), and the influence of culture and religion [24]. Other characteristics such as older age, higher levels of education, and occupational status of women and their husbands, have also been associated with a higher likelihood of a woman being a sole or joint decision-maker regarding contraceptive use [24, 25].

Similar to previously documented findings [8, 26], we found that male partner support for FP and contraception use by their partners covered various aspects of male involvement including communication, decision-making, physical, practical and to some extent emotional support. One of the male respondents reported that he did not communicate with his partner about FP use, and she had solely decided against using a method based on her assumptions. In this instance, communicating with his partner might have provided opportunity to discuss and jointly seek information about various methods. This could have dispelled the partners’ misconceptions and enabled them to make an informed choice. Previous studies have confirmed that when males discuss FP and contraception with their female partners, there is a higher likelihood of uptake and sustained use [27].

Despite challenges posed by the pandemic, men in the study provided practical support, including financial support, transportation, and information seeking about FP commodities as documented in existing literature. However, direct male involvement in promoting FP use was less frequently reported than indirect involvement as only a few men in Oyo state mentioned condom use as the preferred FP method by the couple. This is comparable to findings in existing literature [8].

Importantly, none of the male partners in our study reported outright opposition to FP and none reported being unsupportive of their wives obtaining or renewing contraceptive methods during the pandemic. The high acceptance of methods among the sample could have been because the males who participated in our study were recruited through their partners who sought FP and other contraceptive services in the PHCs. Other possible factors could be the prevailing economic constraints necessitating couples to have the number of children they can cater for as mentioned by some male participants and the effect of previous FP programmes promoting male involvement implemented in the country.

## Conclusion

In summary, our study emphasizes the roles of men in supporting FP and contraceptive use by their partners during the COVID-19 pandemic. While men in Kano predominantly made decisions independently, joint decision-making and decision-making by women alone were more common in Kwara and Oyo states. The support provided by the men played a crucial role in overcoming the challenges of accessing care during the pandemic, such as movement restrictions, unavailability of transportation and vaccine shortages. This support contributed to sustained FP use by women amidst the pandemic’s disruptions. The level of male support we found suggests that previous interventions promoting male involvement in family planning could have impacted men in our study sites. Continued investments in these interventions can further enhance male participation and support for FP and contraception. Efforts should also be made to ensure the availability of FP services during pandemics and emergencies to mitigate the barriers to access.

Our findings have programmatic and policy implications for family planning programmes during health crises. Health education programmes that emphasize the benefits of couple communication and joint decision-making in family planning could be broadcast through various media channels to enhance communication and decision-making among couples. Male partners’ willingness to provide practical support such as transporting their partners to health facilities and drug stores to obtain FP commodities suggests that interventions such as couple counselling both in-person and remotely could further strengthen male involvement. Interventions which promote female autonomy such as confidential remote counselling for female clients, and community-based distribution of self-injectables could be implemented to ensure women continue to have access to FP.

## Limitations

Our study has a few limitations that warrant consideration. Firstly, the male respondents were partners of female clients and may not represent all males, especially those who might not approve of modern family planning methods. Thus, our findings should not be interpreted that all males provided support to their partners during the pandemic.

A few participants refused to participate in the study. We do not think that those who refused were markedly different from those who consented. We could not obtain additional information from them to compare with those who agreed to participate as this would have been a breach of their decision not to participate. Although our findings are robust and were obtained from a range of study participants to provide a broad view, it is essential to interpret them bearing in mind their qualitative nature, which would not represent the views of all women and men in the study sites. However, we made efforts to ensure our findings are trustworthy. The design, implementation and analysis followed guidelines for ensuring trustworthiness of qualitative data to promote credibility, transferability, dependability, and confirmability of our data. To ensure credibility, we engaged researchers with expertise in qualitative research. All researchers contributed to the protocol, promoting dependability of our findings. Our study included diverse respondents knowledgeable about the study topic, such as female FP users, their male partners and females residing within the community. Data collection employed a mix of methods—in-depth interviews and FGDs, facilitating triangulation of responses. Data analysis involved a team approach, with spot checks of transcripts by state leads to ensure transcription was done verbatim. Coding was conducted by a team of coders who developed the codebook in line with recommended guidelines, refining codes as required. Peer debriefing was carried out among the team members who are experts in the study subject. Findings were discussed with the data collection staff, referring to their field notes for clarification during analysis. We did not find any other literature describing the roles that men played in supporting FP uptake by their spouses or female partners during the COVID-19 pandemic thus we could not compare our findings with these. Despite these limitations, we believe our findings are comprehensive and represent the perspectives of our study participants. Our findings also provide valuable insights into decision making for FP use and the roles men played to support family planning use by their female partners in the study sites.

## Data Availability

As the study was qualitative, the transcripts have not been deposited in an open access repository because of the potential risk that information becomes identifiable. The transcripts would however be made available on reasonable request from the corresponding author.

## Abbreviations

COVID: Corona Virus Disease
FGD: Focus Group Discussion
FP: Family Planning
IDI: In-depth Interview
NURHI: Nigerian Urban Reproductive Health Initiative
PHC: Primary Health Care
PPFN: Planned Parenthood Federation of Nigeria
TCI: The Challenge Initiative
WHO: World Health Organization
